# Single nucleotide variants in *Pseudomonas aeruginosa* populations from sputum correlate with baseline lung function and predict disease progression in individuals with cystic fibrosis

**DOI:** 10.1099/mgen.0.000981

**Published:** 2023-04-13

**Authors:** Morteza M. Saber, Jannik Donner, Inès Levade, Nicole Acosta, Michael D. Parkins, Brian Boyle, Roger C. Levesque, Dao Nguyen, B. Jesse Shapiro

**Affiliations:** ^1^​ Department of Microbiology and Immunology, McGill University, Montreal, QC, Canada; ^2^​ Department of Medicine, Research Institute of the McGill University Health Centre, Montreal, QC, Canada; ^3^​ Department of Microbiology, Immunology and Infectious Disease, University of Calgary, Calgary, AB, Canada; ^4^​ Department of Medicine, University of Calgary, Calgary, AB, Canada; ^5^​ Integrative Systems Biology Institute, University of Laval, Québec, QC, Canada; ^6^​ Meakins Christie Laboratories, Research Institute of the McGill University Health Centre, Montreal, QC, Canada; ^7^​ McGill Genome Centre, Montreal, QC, Canada

**Keywords:** AmpliSeq, cystic fibrosis, genomics, machine learning, *Pseudomonas aeruginosa*, within–host diversity, lung function

## Abstract

The severity and progression of lung disease are highly variable across individuals with cystic fibrosis (CF) and are imperfectly predicted by mutations in the human gene CFTR, lung microbiome variation or other clinical factors. The opportunistic pathogen *Pseudomonas aeruginosa* (*Pa*) dominates airway infections in most CF adults. Here we hypothesized that within–host genetic variation of *Pa* populations would be associated with lung disease severity. To quantify *Pa* genetic variation within CF sputum samples, we used deep amplicon sequencing (AmpliSeq) of 209 *Pa* genes previously associated with pathogenesis or adaptation to the CF lung. We trained machine learning models using *Pa* single nucleotide variants (SNVs), microbiome diversity data and clinical factors to classify lung disease severity at the time of sputum sampling, and to predict lung function decline after 5 years in a cohort of 54 adult CF patients with chronic *Pa* infection. Models using *Pa* SNVs alone classified lung disease severity with good sensitivity and specificity (area under the receiver operating characteristic curve: AUROC=0.87). Models were less predictive of lung function decline after 5 years (AUROC=0.74) but still significantly better than random. The addition of clinical data, but not sputum microbiome diversity data, yielded only modest improvements in classifying baseline lung function (AUROC=0.92) and predicting lung function decline (AUROC=0.79), suggesting that *Pa* AmpliSeq data account for most of the predictive value. Our work provides a proof of principle that *Pa* genetic variation in sputum tracks lung disease severity, moderately predicts lung function decline and could serve as a disease biomarker among CF patients with chronic *Pa* infections.

## Data Summary

All amplicon sequencing data generated in this project are deposited in NCBI GenBank under BioProject PRJNA763719: https://www.ncbi.nlm.nih.gov/bioproject/PRJNA763719

**Impact Statement**

Cystic fibrosis (CF) is among the most common, life-limiting inherited disorders, caused by mutations in the CF transmembrane conductance regulator (CFTR) gene. CFTR dysfunction causes impaired mucociliary clearance, leading to chronic airway infections, and a vicious cycle of lung inflammation and damage, resulting in progressive lung disease, the major cause of morbidity and mortality in CF patients. However, the severity of lung disease and the rate of lung function decline are highly variable across CF patients, and cannot be fully explained using existing clinical or host genetic factors. Here we employed machine learning (ML) techniques to establish a link between *Pseudomonas aeruginosa* (*Pa*) within–patient genetic diversity and lung disease severity in a cohort of CF patients with chronic *Pa* infections. Our study provides a proof of principle demonstrating the utility of ML tools for predictive modelling of lung function severity and decline in CF patients using *Pa* genetic diversity data. Our findings show the potential for ML models to identify high-risk CF patients using *Pa* genetic information.

## Introduction

Cystic fibrosis (CF) is an autosomal recessive disorder caused by mutations in the CF transmembrane conductance regulator (CFTR) gene and is the most common lethal Mendelian disease in populations with European ancestry [1]. The resulting lung disease is the major cause of morbidity and mortality in CF patients, with lung failure the most common cause of death [2]. The severity of lung disease and the rate of lung function decline are highly variable across CF patients, and cannot be fully explained by variations in CFTR alleles or other modifier genes [3].

While CF airway infections are polymicrobial, and microbiome diversity has been associated with lung disease severity in many studies ([4–7], *Pseudomonas aeruginosa* (*Pa*) is an opportunistic pathogen found in the majority of adult CF patients and often dominates their airway microbiome [7, 8]. Infection with *Pa* in early life is associated with a greater decline in lung function and mortality [9–11]. Notably, *Pa* airway infections can persist even with highly effective CFTR-modulator therapies [12, 13].

Over the course of chronic CF lung infections, *Pa* undergoes genetic diversification, selection and adaptive evolution, resulting in a genetically and phenotypically diverse *Pa* population within each patient [14–19]. How this pathoadaptation affects the clinical course of CF lung disease remains poorly understood. We therefore focused on examining the association between *Pa* genetic variation and the severity and progression of lung disease in CF patients with chronic *Pa* infections. We hypothesized that within–host genetic variations in *Pa* populations during chronic CF lung infections are associated with lung disease severity (i.e baseline lung function) and subsequent progression (i.e. decline in lung function), as measured by spirometry.

While it is known that within–host mutations can significantly affect the virulence of *Pa* and host responses to *Pa*, previous studies [16, 20–23] have examined the genetic variation of *Pa* across cohorts of CF patients by performing whole-genome sequencing (WGS) of only one or few *Pa* clones isolated from CF sputum samples – an approach that fails to capture the genetic diversity of *Pa* within the lung and is subject to sampling bias. While shotgun metagenomic analysis of CF sputum is increasingly used for microbiome analyses [24], the overwhelming abundance of host-derived DNA in sputum samples still hampers the ability to resolve within-species genetic variation. To overcome these challenges, here we applied a novel amplicon sequencing (AmpliSeq) panel of 209 genes in the *Pa* genome previously known to be involved in the pathoadaptation and pathogenesis of CF infections (Dataset S1, available in the online version of this article). The AmpliSeq platform allows us to estimate single nucleotide variant (SNV) frequencies within the *Pa* population, directly from CF samples, without the need to culture and sequence hundreds of isolates per individual.

We then used several machine learning (ML) approaches to classify lung disease severity (at the time of sampling) and to predict disease progression (after 5 years) based on the SNV frequency data from a cohort of 54 adult CF patients with chronic *Pa* infection. ML has been successfully applied to predict phenotypes from genotype data in other model systems [25]. ML models can explicitly include the interactions and correlations between features (in our case, SNVs), which helps control for confounding factors such as population stratification that may exist in the dataset [26].

Our study provides a proof of principle that the population of *Pa* in CF sputum samples includes bacterial genetic biomarkers that are associated with disease status and could serve to identify individuals at increased risk of rapid lung function decline. Additionally, this work identified genetic variation in *Pa* genes that merit further investigation for their potential roles in the pathogenesis of CF lung disease.

## Methods

### Patient selection, sample and clinical data collection

The Calgary biobank includes frozen whole sputum samples prospectively collected from individuals with CF followed at the Calgary Adult CF clinic from 1998 to 2017, as described previously [27, 28]. A cohort of 104 individuals between the ages of 18 and 22 years with sputum available from the Calgary biobank was previously characterized [27]. For this study, we selected from this cohort all individuals with sputum cultures positive for *Pa* (64 out of 104 patients). Out of these 64 samples, 54 yielded AmpliSeq data of sufficient depth (>10× average depth of coverage of the targeted genes) and were retained for further analysis. Clinical data collected for each patient are outlined in Tables 1 and S1 and include age, gender, body mass index, CFTR genotype, birth cohorts and microbiology (mucoid phenotype, *Pa* relative abundance, microbiome diversity indices).

**Table 1.** Patient clinical data Values show the absolute count, or mean with standard deviation in parentheses where applicable. Baseline lung function is defined as severe when FEVp <60% predicted and mild otherwise. Lung function decline is defined as non-rapid when 5 year FEVp decline <5 % and rapid otherwise. The relative abundance of *Pa*, as well as Shannon and Simpson diversity indices, were computed based on 16S rRNA gene sequencing of the lung microbiome community. Homozygous ΔF508 indicates the counts of individuals with a ΔF508/ΔF508 genotype; others include heterozygotes or other genotypes. Test statistics are the Wilcoxon rank-sum statistic for numerical data [*Pa*, age, body mass index (BMI), Shannon and Simpson indices] and odds ratio for categorical data.

Patient data	Baseline lung function				Lung function decline over 5 years			
	Severe (*n*=27)	Mild (*n*=27)	Test statistic	*P* value	Non-rapid (*n*=31)	Rapid (*n*=23)	Test statistic	*P* value
*Pa* relative abundance	0.59 (0.32)	0.35 (0.32)	2.48	0.01	0.46 (0.33)	0.49 (0.32)	0.3	0.75
Age (years)	19.0 (1.13)	19.4 (1.16)	1.40	0.16	19.2 (1.1)	19.2 (1.25)	0.24	0.80
BMI (kg m^–2^)	19.0 (2.3)	21.4 (2.3)	3.45	0.0005	20.62 (2.9)	19.7 (2.1)	1.18	0.23
Shannon index	1.12 (0.64)	1.21 (0.68)	0.42	0.66	1.31 (0.66)	1.06 (0.65)	1.44	0.24
Simpson index	0.48 (0.26)	0.5 (0.28)	0.23	0.81	0.54 (0.26)	0.45 (0.27)	1.61	0.20
PES (PFGE typing)	15	9	2.5	0.17	16	8	2.0	0.27
Homozygous ΔF508	15	13	1.34	0.78	14	14	0.52	0.28
Not-deceased (Death)	22	25	0.35	0.42	27	20	1.01	1
Male (Gender)	11	9	0.72	0.77	8	12	3.13	0.86
Mucoid	24	23	0.71	1	27	20	0.98	1
Birth cohort years 1978–1984	11	8	0.47	0.49	12	7	1.31	0.25
Birth cohort years 1985–1990	9	11	0.2	0.65	7	13	1.8	0.17

PES, Prairie Epidemic Strain.

As a measure of lung disease severity at the time of sputum collection, we used the spirometric measure of forced expiratory volume in 1 s, percentage predicted (hereafter referred to as ‘baseline lung function’ and noted FEVp), a standard measure of lung function normalized for age, height, and self-identified sex and ethnicity. Baseline lung function was categorized as severe for FEVp <60 % predicted, and mild/moderate for FEVp ≥60 % predicted based on the European Respiratory Society/American Thoracic Society standard lung function interpretations [29]. Long-term lung function decline (hereafter noted as ‘lung function decline’) was measured using the relative rate of FEVp decline per year (determined by subject-specific constructed linear regressions over the 5 years following sputum collection as described by Acosta *et al*. [27]. Lung function decline was categorized as ‘rapid’ when the relative FEVp decline was >5 % per year, and ‘non-rapid’ when ≤5 % per year.

### Sputum DNA extraction and microbiome analyses

Genomic DNA was extracted from a single biobanked sputum sample per patient as previously described [27], and used as template for 16S rRNA gene amplicon and Ion AmpliSeq sequencing. The Prairie Epidemic Strain (PES) genotype, a highly prevalent strain in our study population, was identified by PFGE and/or multi-locus sequence typing (MLST) . For microbiome analysis, bacterial communities in CF sputum and reagent blanks were characterized by amplification and sequencing of the V3–V4 region of the 16S rRNA gene, as previously described [27]. The sequencing reads were then processed to identify operational taxonomic units (OTUs) [28]. Relative *Pa* abundance was determined as the proportion of *Pseudomonas* reads relative to the total number of 16S rRNA gene reads.

### Ion AmpliSeq panel design and sequencing

The AmpliSeq panel targeted 209 *Pa* genes previously implicated in pathogenicity, antimicrobial resistance and within–host pathoadaptation during chronic infection (Data S1). The AmpliSeq primer panel (generated by Life Technologies) was designed by the AmpliSeq Custom Services (White Glove, Thermo Fisher Scientific) to provide high sequencing coverage of the target genes based on the *Pa* PAO1 genome (NCBI accession number: GCA_000006765.1)*,* with 100 % breadth of coverage for 205 genes and >96 % in four genes, based on the tiling of amplicons. Four additional genome assemblies of *Pa* clinical isolates [GCF_004375495.1, GCF_004374685.1, GCF_004374275.1 and the PES genome (NCBI BioProject: PRJNA750451)] were also evaluated along with PAO1 for the optimization of primer design, tiling and pooling to achieve maximal target coverage by the primer panel with minimal misalignments and homology with the human genome.

AmpliSeq libraries were constructed using the Ion AmpliSeq Library kit 2.0 and IonCode barcode set with the following modifications. SparQ magnetic beads (Quantabio) were used for purification, and individual libraries were quantified using the Quant-iT PicoGreen dsDNA Assay Kit (ThermoFisher). Samples were mixed in equimolar proportions and the pooled library (200 pM) was loaded on an Ion Chef for template preparation using HiQ reagents. The P1 v3 chips were sequenced using an Ion Proton sequencer (500 flows) with P1 HiQ sequencing reagents following the manufacturer’s instructions.

### AmpliSeq variant calling

The quality of AmpliSeq sequencing was confirmed using TorrentSuite software (v.5.2; Thermo Fisher Scientific). Raw sequencing reads were trimmed based on a per-base phred quality score cutoff (‘q’ flag) of 18, window size of 1 bp and minimum remaining sequence length (‘l’ flag) of 19 using fastq-mcf (v.1.04.636) [30]. Reads were aligned to the PES genome (CP080405) using BWA MEM and the alignments were sorted and indexed using SAMtools (v.1.9) [31]. Samples with average sequencing depth ≤10× across the target genes were discarded, leaving 54 samples for further analysis. SNVs with minimum mapping quality of 20, minimum base quality of 18 and minimum coverage of 10× were then identified using VarScan 2 [32] and functional consequences of each SNV were inferred using snpEFF (v.2.4.2) [33]. The SNV allele frequencies (ranging from 0 to 1) at each polymorphic site covered by the AmpliSeq panel were used to generate an SNV frequency matrix, with samples as rows and nucleotide positions as columns. For baseline lung function (measured based on FEVp score) and lung function decline (disease progression) prediction analysis, all synonymous variants were filtered out and only non-synonymous variants (including nonsense and missense mutations, frameshift deletions and insertions) were used (Dataset S2). All SNVs (including synonymous sites) were included for population stratification analyses.

### Heritability estimation

Here we define heritability as the variation in disease status that can be explained by genetic variation measured in the AmpliSeq SNV data. We estimated heritability as the average prediction accuracy *R* ^2^ from the elastic net model implemented in the scikit-learn Python package in held-out samples during cross-validation on continuous phenotypes (not binned into mild/severe or rapid/non-rapid). This approach is similar to that described previously by Lees *et al*. [26].

### Bacterial population stratification

Population stratification in *Pa* was evaluated by calculating pairwise Pearson correlation coefficients between sputum samples based on the SNV frequency matrix followed by determination of distinct genome subgroups using hierarchical agglomerative clustering implemented in SciPy [34] and visualized using the python seaborn package [35]. This identified two major subclusters of *Pa,* one of which was significantly enriched in PES strains. To determine if any clinical factors were associated with these subclusters, we used *t*-tests for continuous variables including age, body mass index (BMI), Shannon and Simpson diversity indices and *Pa* abundance in the sputum sample. For binary variables including PFGE typing (PES or not), gender, host CFTR genotype, death, mucoid presence/absence status, baseline lung function and lung disease progression (lung function decline), we used a Fisher Exact test. A Chi-square test was used for the multi-categorical birth cohort factor.

### Feature selection and training predictive model of lung function

In an ML context, a feature is defined as an individual measurable characteristic of an observed phenomenon. In this study, the features considered are *Pa* genetic variants (SNV frequencies) identified by the AmpliSeq panel and the clinical factors linked to the study patients (Table S1). To reduce the dimensionality of the dataset (i.e. to reduce the ratio of features to sample size), a feature selection approach was applied using nested cross-validation in three steps.

1. Outer loop: data were split into 80 % training (43 samples) and 20 % testing (11 samples) for 20 resamplings (folds) using a stratified shuffled split function implemented in sklearn [36]. This method ensures no overlap between train and test dataset in each fold but samples in the training dataset may overlap across folds. Stratification was performed along labels in each phenotype (i.e. baseline lung function and lung function decline) to keep the ratio of cases to controls in train and test datasets similar in each fold.

- Inner loop: from the training dataset within each outer loop, samples were randomly bootstrapped (*n*=43, sampling with replacement) and feature importance (i.e. scores assigned to each input feature indicating the relative importance of the feature when making a prediction) were estimated using the lightGBM [37] gradient boosting ensemble method implemented in feature-selector v1.0.0 [38] for 50 folds. Feature-selector parameters were set as: n estimators=1000, learning_rate=0.05 and early_stopping=True.
- Features with an importance value of zero averaged across 50 inner loops were discarded. Each of four ML models (i.e. logistic regression, SVM, random forest and XGBoost) were independently trained on a dataset of selected features and performance of the trained model was measured by estimating ROC-AUC on the test dataset ensuring no data leakage between training and testing datasets.

2. Feature importance values estimated in the 20 outer loops were averaged and the set of features required to obtain 99 % cumulative relative importance were retained to perform prediction analysis. The remaining features were filtered out.

3. ROC-AUCs of models were estimated by averaging the ROC-AUCs over the 20 outer loops.

To prevent bias or overfitting to the training data, we performed feature selection within the inner cross-validation such that no information from the training data is leaked to the held-out samples. In other words, to find the optimal coefficient values for each feature, we used inner cross-validation and set the feature importance to 0 for the features that did not contribute to the classification of the data in the test dataset, thereby preventing any leakage of information between training and testing datasets.

Four ML models were used in this study: logistic regression with l2 regularization, extreme gradient boosting implemented in XGBoost [39], ensemble decision trees implemented in random forest [40] and linear support vector machine (SVM) with linear kernel implemented in the sklearn package with default hyperparameters. Model performance was evaluated using six metrics: (1) area under the receiver operating characteristic (AUROC), accuracy (number of correct predictions/total number of predictions); (2) precision (True Positives/(True Positives+False Positives)); (3) recall (True Positives/(True Positives+False Negatives)); (4) F1 score (2*(Precision*Recall)/Precision +Recall); (5) Accuracy; and (6) balanced accuracy (bACC), the average of recall obtained on each class (i.e. severe/mild for baseline lung function and rapid/non-rapid for lung function decline).

To evaluate the statistical significance of the prediction performances (AUROC scores) obtained by ML models in comparison with random expectations, a non-parametric permutation test [36] was performed using 100 rounds of label switching followed by feature selection and model training (as done for real data) and estimating empirical *P*-values (i.e. the chance that the observed AUROC scores obtained using the data could be obtained by chance alone). The enrichment of predictor SNVs across functional gene categories relative to the total genes in the AmpliSeq panel was assessed using Fisher’s exact test with a family-wise error rate of 0.05 adjusted for multiple testing using the Bonferroni method.

### Code availability

All computer code used to conduct the AmpliSeq data analysis, including ML methods, are available at GitHub at the following link: https://github.com/Morteza-M-Saber/Cystic_fibrosis_ML_analysis/

## Results

We studied a previously described and well-characterized cohort of young adult CF patients aged 18–22 years with chronic *Pa* infection [27]. After filtering for AmpliSeq sequencing quality, we excluded 10 patients with low coverage of *Pa*, leaving 54 patients for further analysis. The clinical and demographic characteristics of all 54 patients are summarized in Table 1, and the excluded patients were not apparent outliers in their clinical profiles. From the filtered sequence data, we identified SNVs within the 209 genes represented in the AmpliSeq panel and estimated the frequency of each SNV within each patient sputum sample. In total across the 54 patient samples, we identified 7867 synonymous and 4452 non-synonymous SNVs (Dataset S2). All variants were used for population stratification analysis and only non-synonymous SNVs were used to train ML models.

We first estimated the heritability of baseline lung function (FEVp score) and lung function decline over 5 years. Baseline lung function had a heritability of 0.30 [95 % confidence interval (CI): 0.23–0.37], indicating a significant genetic component from the AmpliSeq data. In contrast, the heritability of lung function decline could not be estimated due to a poor fit of the elastic net model (mean *R* ^2^ across cross-validation folds <0). We therefore expect lung function decline to be challenging to predict from AmpliSeq data. Considering baseline and future lung disease as continuous factors, we found that AmpliSeq data explain significant variance in baseline lung function (elastic net explained variance regression score=0.41; 95 % CI: 0.37–0.46) but not in lung function decline (explained variance ~0). Due to these relatively poor model fits on continuous phenotypes, we binned each measure of disease severity into discrete, clinically relevant categories: (1) severe or mild baseline lung function, and (2) rapid or non-rapid lung function decline over 5 years (see Methods). Both measures of lung disease severity were binned based on clinically accepted threshold values [29]. As described below in detail, ML models were able to classify these discrete disease categories significantly better than random. We therefore proceeded with these discrete categories for subsequent analyses.

### Stratification in the *Pa* population

**Fig. 1.**
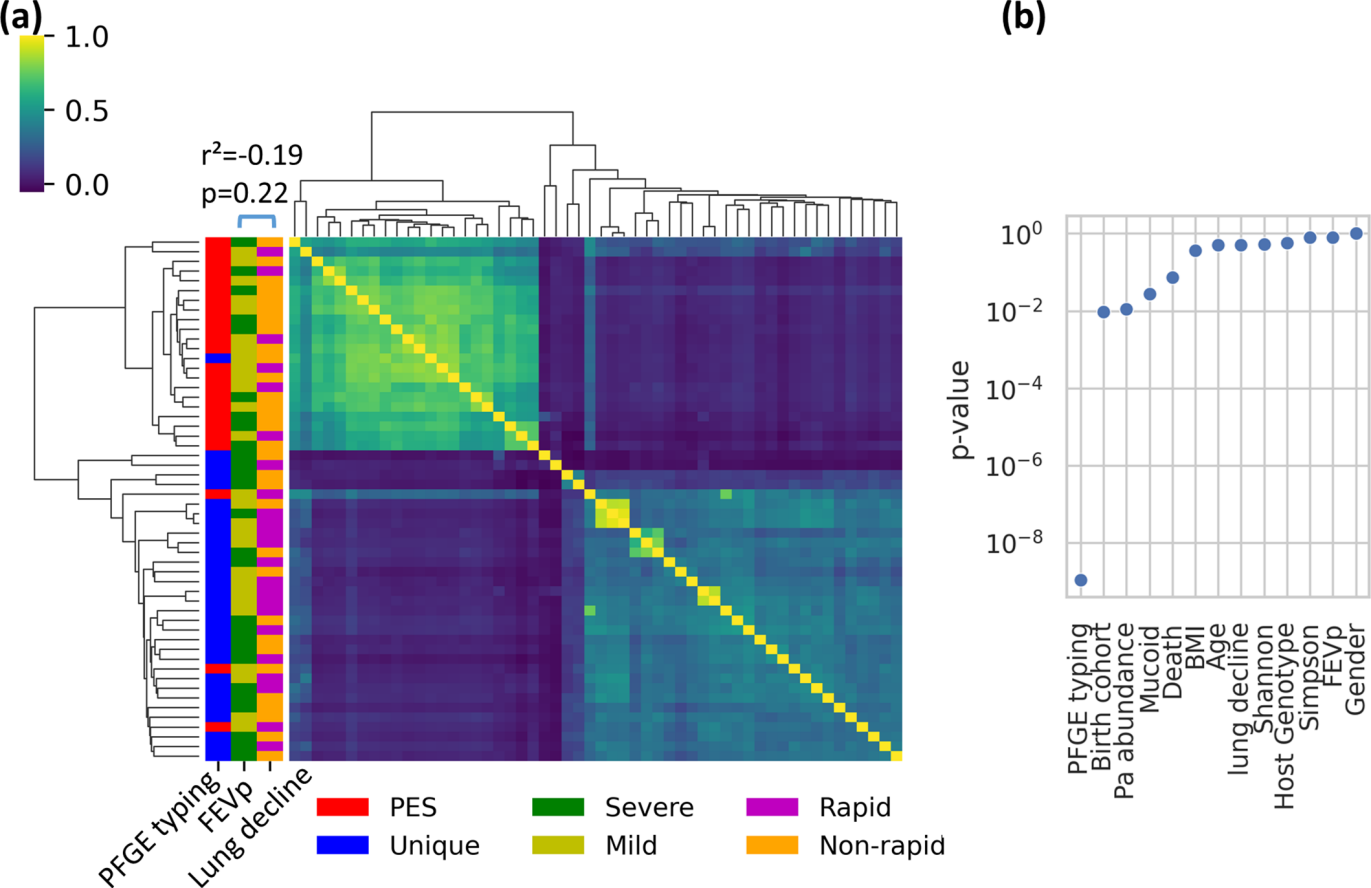
*Pa* populations are stratified into two genetic clusters, neither of which is associated with baseline lung function (FEVp) or lung function decline. (**a**) Heatmap showing correlations in within–host *Pa* SNV frequencies between pairs of sputum samples. Strong correlations are in yellow; weak correlations in blue. Rows and columns (samples) are ordered by hierarchical clustering. Distribution of baseline lung function measured by FEVp score (27 Severe and 27 Mild individuals), lung function decline (23 Rapid and 31 Non-rapid individuals) and PFGE typing (25 PES and 29 Unique) are presented on the *y*-axis. Baseline lung function and lung function decline over 5 years are not significantly correlated (Pearson *R* ^2^ score=−0.19, *P*=0.22). (**b**) *P*-values for the association between clinical data and genetic clusters are determined by *t*-tests for numerical data and Chi-square tests for categorical data (Methods). Only the association between PFGE type (PES or non-PES) is significantly associated with the genetic clusters in (a) (*P*<0.0045 after Bonferroni correction for multiple tests).

We quantified the extent of *Pa* population stratification, which can be problematic if there are clonally related genetic clusters that are confounded with the lung disease outcomes of interest. If a particular genetic cluster or lineage is associated with lung disease severity, it then becomes difficult to pinpoint the SNVs associated with disease because all mutations (whether related to disease or not) in a cluster are correlated with each other. We know a priori based on PFGE typing that our dataset contains a highly prevalent lineage of *Pa* [called Prairie Epidemic Strain or PES; sequence type (ST)-192; Table 1] suspected to be associated with severe lung disease [41]. We confirmed this by hierarchical clustering of the *Pa* AmpliSeq data (*n*=12 319 SNVs, including both synonymous and non-synonymous variants), which revealed two apparent genetic clusters (Fig. 1a), one of which was strongly associated with the PES lineage (Fisher exact test, odds ratio=168.0, *P*=1.1e-09; Fig. 1b). The few observed exceptions (i.e. three PES samples grouped with unique PFGE types; Fig. 1a) could be due to mixed infections or sufficient within–host diversification to obscure the genetic signal of PES ancestry. The two major *Pa* genetic clusters were also weakly associated with the birth cohort (Chi-square test, *P=*0.0095; not significant after multiple test correction; Fig. 1b) which is probably due to unequal prevalence of PES across the time periods where cohorts were recruited (Table S1). No other clinical factor was significantly associated with either genetic cluster (Fig. 1b). Importantly, neither cluster is correlated with either baseline lung function (Fisher exact test, *P*=0.81) or lung function decline (Fisher exact test, *P*= 0.51) (Fig. 1b), indicating that these disease outcomes are unlikely to be confounded by *Pa* population stratification, and that finer-grained predictive modelling is warranted. We also noted that lung disease progression over 5 years (lung function decline) is not significantly correlated with baseline lung function at sample collection (Fig. 1a).

### Genetic and clinical features associated with baseline lung function and lung function decline in CF patients

**Fig. 2.**
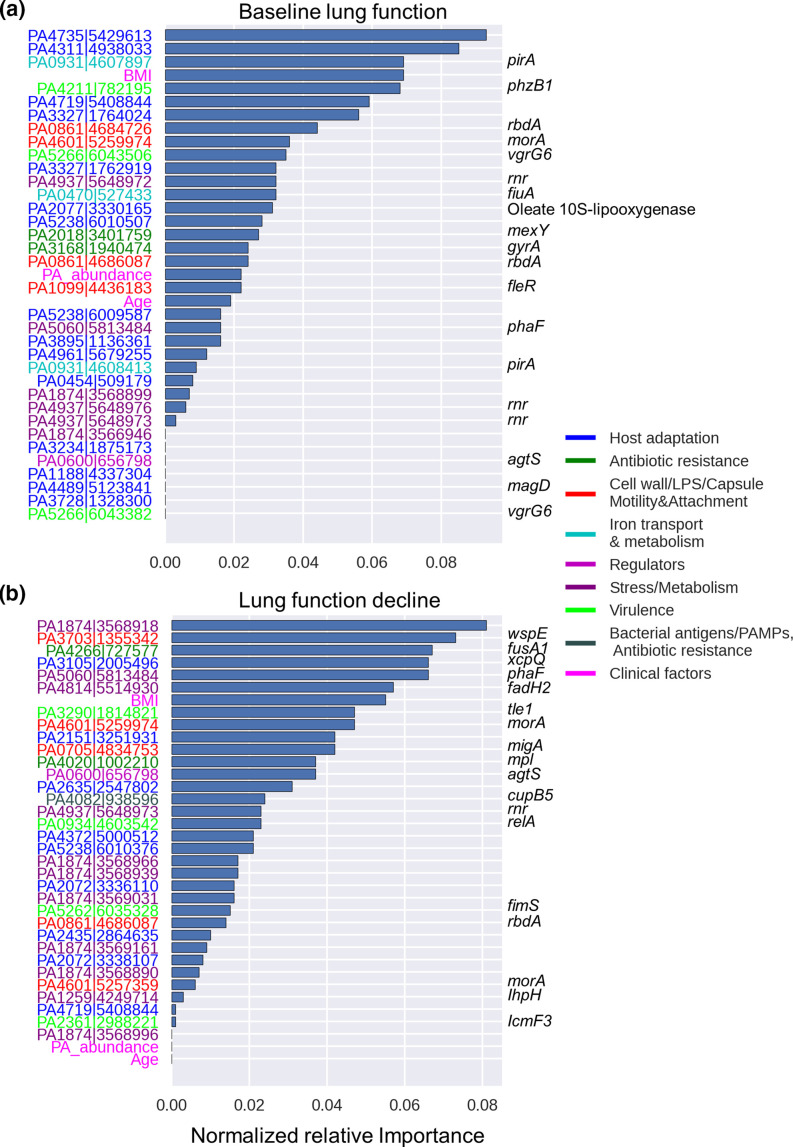
*Pa* genes and clinical factors selected as predictive features of baseline lung function and lung function decline. Normalized importance of genomic and clinical data that contribute to 99 % cumulative relative importance for the prediction of (**a**) baseline lung function at time of sample collection and (**b**) risk of 5 year progression (lung function decline). On the *y*-axis, gene identifiers (locus tag|chromosome location based on PES genome) are colour-coded based on their functional classification. Named genes are shown on the right, when available.

A common challenge in predicting outcomes from sequence data is the sparsity of the data, that is the relatively few available samples compared to the large number of genetic markers (called ‘features’ in ML context). To resolve this problem, feature selection can be used to remove non-informative features (i.e. SNVs and clinical factors) and focus only on the most predictive ones [42–45]. We used a nested cross-validation approach for feature selection based on ensemble gradient boosting (see Methods). Out of 4452 non-synonymous SNVs and 11 clinical factors considered, our model selected only 34 SNVs (hereafter called predictor SNVs) and three clinical factors (age, BMI and *Pa* relative abundance in the sputum microbiome) that account for 99 % of the cumulative feature importance (Fig. 2). This means that a minimal set of SNVs and clinical factors provides 99 % of the information used in predicting baseline lung function at the time of sampling (Fig. 2a). An equivalent analysis for lung function decline after 5 years identified 33 predictor SNVs and the same three clinical factors that contributed to 99 % of the cumulative feature importance (Fig. 2b). Including the number of polymorphic SNVs (within–sample frequency >0 and <1) to the models did not improve predictions nor was it selected as an important feature, suggesting that simple measures of within–patient diversity have limited predicted value. For both baseline lung function and future lung function decline, the phenotype is not simply predicted based on the presence/absence of each SNV, but rather on more subtle information about SNV allele frequencies within patients. In other words, predictive SNVs occur at a range of frequencies, with only 223 being clustered around 0 or 1 (Fig. S1).

The three selected clinical factors associated with both baseline lung function and lung function decline are BMI, *Pa* relative abundance from 16S rRNA gene amplicon sequence data from a previous study of the same cohort [27] and age (Fig. 2). Multiple studies have shown an association between poor lung function and low BMI [13, 46, 47], high abundance of *Pa* [5] and age [5, 48]. As expected, *Pa* relative abundance also showed a strong negative correlation with Shannon and Simpson microbiome diversity indices (Fig. S2), indicating that *Pa* abundance can be considered as a proxy for lung microbiome diversity in our dataset. However, Shannon and Simpson diversity indices were not selected as predictive features in our model, consistent with a previous study [48]. This suggests that, even if low microbiome diversity indices are associated with CF disease progression, the low diversity is probably driven by the dominance of key pathogens such as *Pa.* The recovery of previously known clinical determinants of lung function in CF patients supports the reliability of our feature selection approach.

**Fig. 3.**
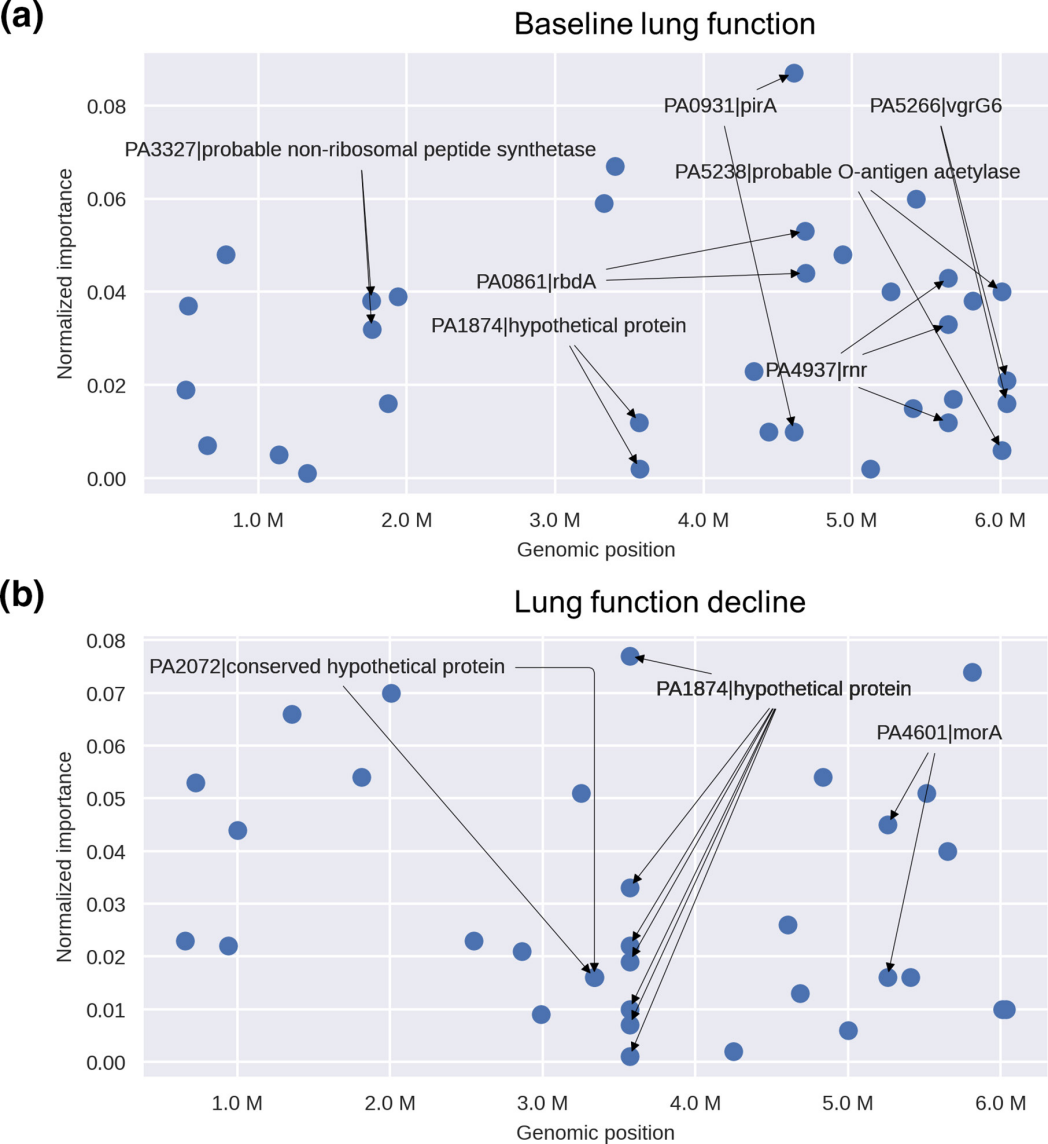
Genomic locations and importance of genes containing predictor SNVs. (**a**) Genes containing SNVs predictive of baseline lung function. The *x*-axis shows nucleotide positions across the *Pa* reference genome. (**b**) Genes containing SNVs predictive of lung function decline. Genes including multiple SNVs are shown with arrows. Note that the two arrows from PA2072 point to two different nearby SNVs in the same gene.

To interpret the possible roles of *Pa* SNVs in CF lung disease, we classified the known or predicted function of genes containing predictor SNVs (hereafter called predictor genes) into functional categories manually curated based on existing literature. The predictor SNVs with the highest weighted importance for both baseline lung function and lung function decline outcomes are located within genes that play a role in seven functional categories (Table 2). The distribution of predictor genes is generally similar to the distribution of gene functions included in the AmpliSeq panel (Fig. S3). However, the predictor genes for baseline lung function are enriched in iron transport and metabolism (13.4 % in baseline lung function predictor genes vs. 1.4 % in the AmpliSeq panel, *P=*0.00018; Fig. S3). The genes encoding the ferric enterobactin receptor (PirA) and the ferrichrome receptor (FiuA) respectively account for 8.9 and 3.7 % of the total normalized importance for baseline lung function (Fig. 2a), and *pirA* contains multiple predictor SNVs (Fig. 3a). In contrast, the predictor genes for lung function decline are enriched in stress/metabolism functions (33.6 % in lung function decline predictor genes vs. 13.4 % in the AmpliSeq panel, *P*=0.002; Fig. S3). Notably, the hypothetical protein PA1874 accounts for 16.9 % of the total normalized importance for prediction of lung function decline and includes seven out of 33 predictor SNVs (Figs 2b and 3b), as well as two predictor SNVs for baseline lung function (Fig. 3). This hypothetical protein has also been shown to play a role in resistance of *Pa* to multiple antibiotics [49]. The PA4937 gene, which encodes an RNase R exoribonuclease, also contains multiple SNVs predictor genes of both baseline lung function and lung function decline (Figs 2a and 3a). The two genes PA0861 (*rbdA*) and PA4601 (*morA*), which encode regulators involved in bacterial motility and biofilm formation, are also predictor genes for both baseline lung function and lung function decline (Fig. 2).

**Table 2.** Functional classification of predictor genes used for prediction of baseline lung function and lung function decline

	Baseline lung function predictor genes	Lung function decline predictor genes	Shared genes
Host adaptation	PA0454|*conserved hypothetical protein* PA1188|*hypothetical protein* PA2077|*oleate 10S-lipoxygenase* PA3234|*probable sodium:solute symporter* PA3327|*probable non-ribosomal peptide synthetase* PA3728|*hypothetical protein* PA3895|*probable transcriptional regulator* PA4311|*conserved hypothetical protein* PA4489|*magD* PA4735|*hypothetical protein* PA4961|*hypothetical protein*	PA2072|*conserved hypothetical protein* PA2151|*conserved hypothetical protein* PA2435|*probable cation-transporting P-type ATPase* PA2635|*hypothetical protein* PA3105|*xcpQ* PA4372|*hypothetical protein*	PA4719|*probable transporter* PA5238|*probable O-antigen acetylase*
Antibiotic resistance	PA2018|*mexY* PA3168|*gyrA*	PA4020|*mpl* PA4082|*cupB5* PA4266|*fusA1*	
Cell wall, LPS, capsule, motility and attachment	PA1099|*fleR*	PA0705|*migA* PA3704|wspE PA4082|*cupB5*	PA0861|*rbdA* PA4601|*morA*
Iron transport and metabolism	PA0470|*fiuA* PA0931|*pirA*		
Regulators			PA0600|*agtS*
Stress/metabolism		PA1259|*lhpH* PA4814|*fadH2*	PA1874|*hypothetical protein* PA4937|*rnr* PA5060|*phaF*
Virulence	PA4211|*phzB1* PA5266|*vgrG6*	PA0934|*relA* PA2361|*icmF3* PA3290|*tle1* PA5262|*fimS*	

LPS, lipopolysaccharide.

#### Predicting lung disease severity and progression in individuals with CF using genetic and clinical factors

**Fig. 4.**
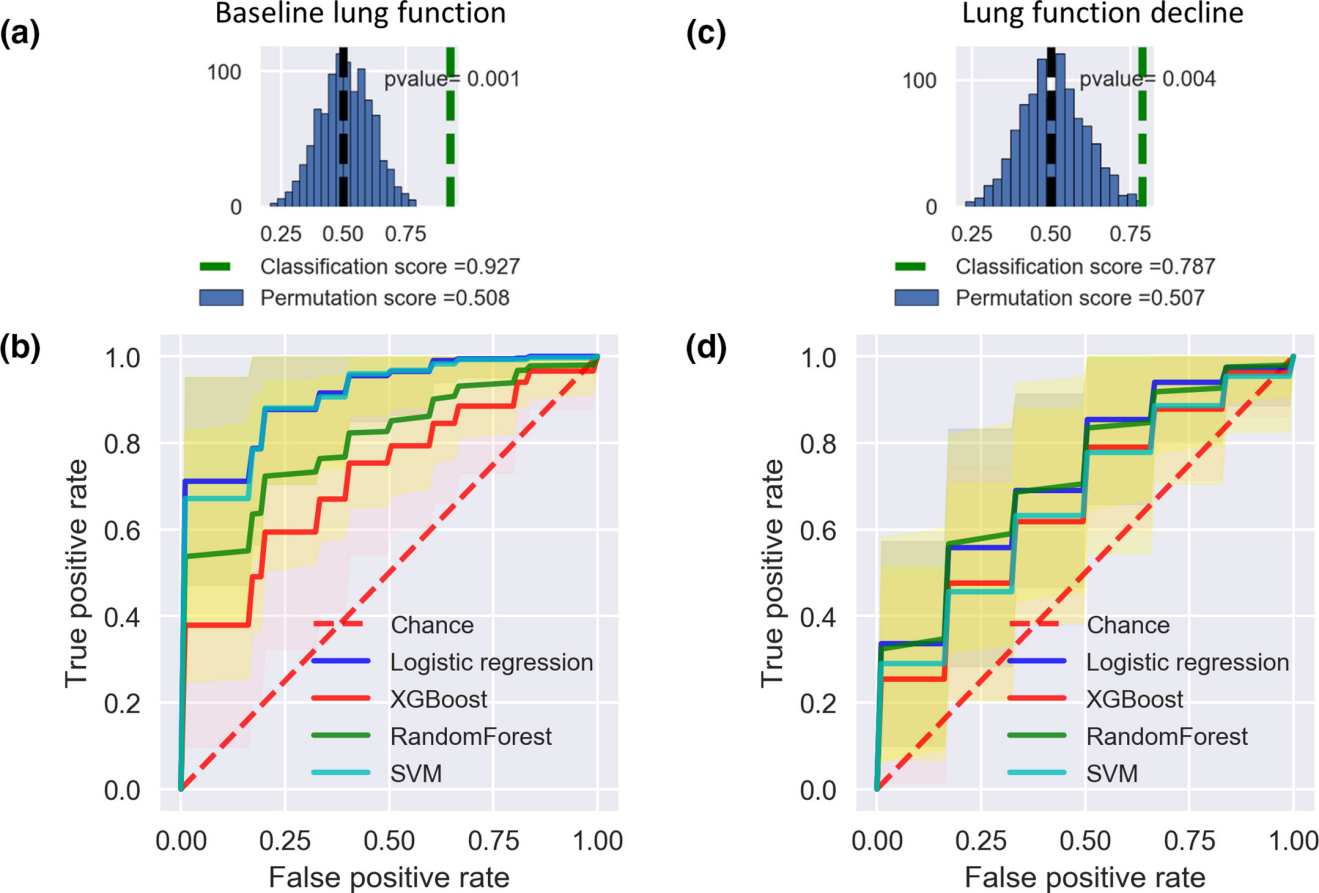
Predictive models of baseline lung function and lung function decline perform significantly better than expected by chance. (a) Classification score of baseline lung function using logistic regression (green dashed line) is significantly higher than expected based on permuted data (mean shown in black dashed line). (b) Average AUROC scores of different ML models to predict baseline lung function, compared to the random expectation (permuted sample labels). Shading indicates the 95 % confidence interval. (c) Classification score of lung function decline using logistic regression compared to permuted data. (d) Average AUROC scores for lung function decline prediction.

We used a nested cross-validation approach to compare the performance of four L models including l2-regularized logistic regression, support vector machines (SVM), random forests, and extreme gradient boosting (XGBoost) using the AUROC curve and other standard metrics (see Methods). To prevent data leakage (i.e. information from outside the training dataset being used to create the prediction model), feature selection was performed on a training dataset and the performance of the trained model was estimated on a completely independent test dataset. It should be noted that while an ensemble light gradient boosting model was used for feature selection (see Methods), we did not use it for predictive modelling to avoid overfitting. Of the ML methods tested, logistic regression had the best predictive performance for both phenotypes (Table 3, Fig. 4). Logistic regression is a simple classification model, which makes it reasonably robust against overfitting [50]. Cross-validation showed that logistic regression was the most accurate and precise model for both baseline lung function, with an average AUROC score of 0.87 (95 % CI, 0.84–0.90), and for lung function decline, with a score of 0.74 (95 % CI: 0.71–0.78; Tables 3 and S2). The second best method was SVM, another type of linear model (Table S2). Across all models, the baseline lung function phenotype was more accurately predicted than lung function decline, consistent with predictions becoming more uncertain further into the future (Tables 3 and S2). Importantly, all models could predict both phenotypes significantly better than expected by chance (compared to a permutation test using data with shuffled outcome labels; Figs 4a, c and S4).

**Table 3.** Performance of logistic regression in predicting baseline lung function and lung function decline using genomic data only, or a combination of genomic and clinical data See Methods for descriptions of the performance metrics.

		Genomic data (95 % CI)	Genomic and clinical data (95 % CI)
Baseline lung function	AUROC	0.87 (0.84,0.9)	0.92 (0.84,1)
	bACC	0.81 (0.78,0.84)	0.83 (0.72,0.94)
	Accuracy	0.81 (0.78,0.84)	0.83 (0.72,0.94)
	F1	0.81 (0.78,0.83)	0.83 (0.72,0.94)
	Precision	0.83 (0.81,0.86)	0.84 (0.73,0.94)
	Recall	0.81 (0.78,0.84)	0.83 (0.72,0.94)
Lung function decline	AUROC	0.74 (0.71,0.78)	0.79 (0.7,0.88)
	bACC	0.63 (0.59,0.66)	0.66 (0.59,0.74)
	Accuracy	0.64 (0.6,0.67)	0.67 (0.6,0.75)
	F1	0.62 (0.58,0.65)	0.66 (0.58,0.74)
	Precision	0.65 (0.61,0.69)	0.69 (0.6,0.78)
	Recall	0.64 (0.6,0.67)	0.67 (0.6,0.75)

Clinical factors have been previously used to predict lung disease progression in CF patients [51]. We therefore assessed if integrating clinical factors could improve upon the predictions based on *Pa* AmpliSeq data alone. Including the three clinical factors identified by feature selection (BMI, age and *Pa* relative abundance) in our predictive models led to modest performance increases (~5 % increase in AUROC) for both baseline lung function and lung function decline outcomes across the four ML models considered (Tables 3 and S2). Using clinical factors alone was always inferior to AmpliSeq data to classify baseline lung function, and was unable to predict lung function decline better than a random expectation (Fig. S5). We conclude that, while these clinical factors are useful, most of the predictive power comes from the *Pa* genetic data.

Lack of generalizability is one of the main limiting factors for the translation of prediction models into clinically useful diagnostics. ML models often have low generalizability (i.e. ‘overfit’) in scenarios where the model performs well on the dataset used to train the model but fails to achieve similar prediction accuracy on new data. We plotted learning curves to assess how ML (using logistic regression) predictions improved by training on more data [52]. We found that the performance difference between training and testing data decreases as sample size increases (Fig. S6). There were no major differences in prediction accuracy of training and testing datasets (Fig. S6), which suggests the model does not suffer from significant overfitting. We also noted that cross-validation scores for both baseline lung function and lung function decline models continued to increase for the testing dataset as more data were used for model training (Fig. S6), which suggests the model could be further improved with more data.

## Discussion

Considering the critical role of *Pa* in CF-related morbidity and mortality, here we established a link between *Pa* within–host genetic diversity and CF lung disease severity in a cohort of young adults with chronic *Pa* infections. Despite a modest sample size, our study provides a proof of principle demonstrating the utility of ML models for predictive modelling of lung function severity and decline in CF patients using bacterial genetic and clinical data. Although our models do not appear to be significantly overfitted, fully validating their predictive performance will require independent cohorts. We also identified potential genetic biomarkers associated with lung disease severity. Overall, our findings provide evidence that ML models can identify CF individuals at high risk for poor *Pa* infection outcomes using *Pa* genetic data.

Our work is based on a subset of samples from a previously described cohort study that identified dominance of *Pa* in the sputum microbiome (and the resulting reduction of community diversity) as a predictor of lung function decline in a cohort of young CF adults [27]. Here we focused on a subset of patients with a lung microbiome dominated by *Pa.* While these patients are already at increased risk of lung disease, we found that the severity of disease at the time of sampling and 5 years into the future could be predicted based on genetic variation within the infecting *Pa* population. Even in this patient cohort in which *Pa* was always present, we confirmed that *Pa* relative abundance is associated with disease severity and progression – although it is a less important predictor than many SNVs within the *Pa* genome. This suggests that genetic variation in dominant pathogens can significantly complement and improve upon predictions of disease status based on the microbiome. Along these lines, another recent study showed that the *Pa* genomic data could predict pathogenicity in mouse models [53].

In addition to variation in the host genome, the polymicrobial community inhabiting the CF lung has been identified as an important modifier of disease progression. Numerous studies of the lung microbiome have shown an association between decreasing microbial community diversity and worsening lung function [4–7, 54], as well as progression to end-stage lung disease [27]. However, microbiome diversity may have limited predictive value as there is high interpersonal variability in lung microbiomes [6], and a large number of adult CF individuals have microbiomes dominated by pathogens such as PA. Zhao *et al*. [55] recently showed that a combination of microbiome data and clinical metadata improved predictive performance compared to either data type alone. Our results suggest that genetic diversity within key pathogens such as *Pa* could complement or even supersede microbiome community diversity for predicting clinical outcomes in specific patient subsets.

Limitations of our study include a relatively small sample size of patients (*N=*54) from a single cohort. As such, we consider our work a proof of concept that could be improved upon in larger cohorts. Indeed, learning curves showed that predictive accuracy is likely to improve with more samples. Although we performed nested cross-validation by subsampling our 54 patients for model training and testing, the model should ideally be tested on a completely independent cohort to assess its real-world predictive value. Other recent studies have suggested that standard cross-validation techniques can overestimate predictive accuracy due to strong genetic linkage across bacterial genomes [26, 56, 57]. *Pa* has a relatively high recombination rate [58] which should reduce the confounding effects of linkage. Importantly, the AmpliSeq data quantify within–patient genetic diversity which does contain traces of ancestry (e.g. PES vs. non-PES lineages) but should also be enriched in *de novo* mutations which are unlinked to deep-branching genomic backgrounds. Future work could attempt to disentangle *de novo* mutations from co-infection with different lineages, thereby determining which predictive features are lineage-associated. Nevertheless, we used a high number of cross-validation folds (*k*=20 for the outer loop and *k*=50 for the inner loop) relative to the sample size of 54 to help reduce the overestimation of accuracy. Using high values of *k* is similar to ‘leave-one-strain-out’ validation, which can be less prone to accuracy inflation [56]. Regardless, the accuracy and generality of our results will require replication in independent cohorts. Despite these limitations, our models made significantly better predictions than expected by chance. As expected, predicting lung function decline 5 years into the future proved more challenging than doing so at the time of sampling. These results provide a key first step toward clinical diagnostics of patients most at risk of lung function decline.

As with any genotype–phenotype association method, our approach does not fully guarantee causal relationships, and rather points to candidate genes. Further experimental testing is therefore required to determine whether *Pa* SNVs play a causal role in lung function decline, or simply serve as useful biomarkers. Regardless, we were able to pinpoint SNVs in several genes of interest. This was feasible because the strong population stratification of *Pa* into PES and non-PES lineages was fortunately not associated with the disease outcomes of interest. This allowed us to identify SNVs in several genes that provided independent biomarkers of disease.

Several genes containing SNVs predictive of disease status and progression were identified as candidates for further investigation. For example, baseline lung function predictor SNVs are enriched in genes involved in iron transport and metabolism. The AmpliSeq panel only included three iron-related genes, of which two (*pirA* and *fiuA*) contained SNVs associated with baseline lung function. Updated AmpliSeq panels or whole-genome sequencing, along with targeted experimental studies, could be used to test the hypothesis that variation in these genes plays a role in disease progression. Multiple studies have shown competition for iron to be key for the survival and virulence of many of the pathogens that reside in the CF lung, including *Pa* [59, 60]. We also found that SNVs predictive of lung function decline are enriched in genes involved in stress/metabolism. Notably, the gene PA1874 includes seven predictor SNVs comprising 16.9 % of the total feature importance for lung function decline, and two predictor SNVs for baseline lung function prediction, suggesting its general importance in disease severity and progression in CF patients. PA1874 encodes a multidrug efflux pump involved in biofilm-dependent resistance to antibiotics including tobramycin, gentamicin and ciprofloxacin [49, 61] and could be a potentially promising biomarker of CF disease severity, which merits further investigation.

Among the set of clinical factors studied, BMI, *Pa* abundance and age were identified as important predictors of both baseline lung function and lung function decline. These are all known risk factors for CF disease severity and progression [5, 46–48]. By including these features in our prediction models, we noted a moderate increase across all the measured metrics relative to using only AmpliSeq data. These results are in line with previous studies showing the improvement of ML-based phenotype prediction by adding relevant clinical data [53, 62]. We note that clinical factors only modestly improved the performance of the models (~5 %), highlighting the rich information and predictive value of the *Pa* AmpliSeq data alone.

In summary, our study demonstrates that SNVs in the *Pa* genome, identified by ML models, can be powerful predictors of lung disease severity and progression in CF patients with chronic *Pa* infections. Even though this disease outcome is affected by multiple microbial, host genetic and environmental factors, *Pa* SNVs add complementary predictive value. With additional genetic and clinical data, our ML model could be further fine-tuned and eventually used as a biomarker to pre-emptively identify individuals with CF at high risk for more aggressive observation and treatment.

## Supplementary Data

Supplementary material 1

Supplementary material 2

Supplementary material 3
