# Supplementary material for: Single nucleotide variants in Pseudomonas aeruginosa populations from sputum correlate with baseline lung function and predict disease progression in individuals with cystic fibrosis
Source: Microb Genom. 2023 Apr 13;9(4):mgen000981. doi: 10.1099/mgen.0.000981 (PMC10210962; doi:10.1099/mgen.0.000981)
Supplement: Supplementary material 1 [file mgen-9-981-s001.pdf]

Supplementary Tables and Figures

Supplementary Table 1. Definition of clinical and microbial factors evaluated for prediction of lung function severity and progression in CF patients.

Clinical metadata	Definition
PFGE typing	*Pa* genotype assessed by Pulsed-Field-Gel electrophoresis (Prairie Epidemic Strain (PES)/ Unique).
Birth cohort	Patients grouped according to the data of birth.
Pseudomonas abundance	Relative abundance of *Pa* in sputum based on 16S rRNA gene sequencing (*Pseudomonas* vs total bacterial load reads).
Mucoid	Presence or absence of mucoid colony morphology in dominant *Pa* strain (cultured from patient sputum).
Death	Recorded until end of data collection in 2017 (Deceased/ Not-deceased).
BMI	Body Mass Index.
Age	Age at time of sputum collection.
Host Genotype	CFTR genotype (ΔF508 homozygous/ΔF508 heterozygous and other types).
Biologic sex at birth	Male or Female
Shannon	Shannon diversity index computed based on 16S rRNA gene amplicon sequencing data of the sputum microbiome.
Simpson	Simpson diversity index computed based on 16S rRNA gene amplicon sequencing data of the sputum microbiome.

Supplementary Table 2. Performance metrics of different ML models for predicting baseline lung function (FEVp) and lung function decline.

		Genomic data (95% CI)				Genomic and clinical data (95% CI)			
		**LR**	**SVC**	**RF**	**XGB**	**LR**	**SVC**	**RF**	**XGB**
**FEVp**	**AUROC**	0.87 (0.84,0.9)	0.87 (0.84,0.89)	0.76 (0.72,0.79)	0.66 (0.62,0.7)	0.92 (0.84,1)	0.90 (0.85,0.99)	0.83 (0.7,0.94)	0.77 (0.62,0.88)
	**bACC**	0.81 (0.78,0.84)	0.79 (0.76,0.81)	0.69 (0.66,0.73)	0.61 (0.58,0.65)	0.83 (0.72,0.94)	0.84 (0.75,0.93)	0.77 (0.67,0.87)	0.7 (0.6,0.8)
	**Accuracy**	0.81 (0.78,0.84)	0.79 (0.76,0.81)	0.69 (0.65,0.73)	0.61 (0.57,0.65)	0.83 (0.72,0.94)	0.84 (0.75,0.93)	0.76 (0.67,0.86)	0.7 (0.6,0.8)
	**F1**	0.81 (0.78,0.83)	0.78 (0.75,0.81)	0.68 (0.65,0.72)	0.6 (0.56,0.64)	0.83 (0.72,0.94)	0.83 (0.74,0.93)	0.76 (0.66,0.86)	0.69 (0.59,0.79)
	**Precision**	0.83 (0.81,0.86)	0.81 (0.79,0.84)	0.71 (0.67,0.75)	0.63 (0.59,0.68)	0.84 (0.73,0.94)	0.85 (0.76,0.93)	0.8 (0.7,0.9)	0.75 (0.64,0.87)
	**Recall**	0.81 (0.78,0.84)	0.79 (0.76,0.81)	0.69 (0.65,0.73)	0.61 (0.57,0.65)	0.83 (0.72,0.94)	0.84 (0.75,0.93)	0.76 (0.67,0.86)	0.7 (0.6,0.8)
**Lung Decline**	**AUROC**	0.74 (0.71,0.78)	0.68 (0.63,0.73)	0.7 (0.66,0.75)	0.62 (0.58,0.66)	0.79 (0.7,0.88)	0.69 (0.58,0.81)	0.77 (0.63,0.85)	0.66 (0.57,0.76)
	**bACC**	0.63 (0.59,0.66)	0.58 (0.55,0.61)	0.58 (0.54,0.62)	0.58 (0.55,0.61)	0.66 (0.59,0.74)	0.59 (0.56,0.62)	0.63 (0.59,0.66)	0.58 (0.54,0.62)
	**Accuracy**	0.64 (0.6,0.67)	0.61 (0.58,0.63)	0.59 (0.56,0.63)	0.6 (0.56,0.63)	0.67 (0.6,0.75)	0.61 (0.58,0.64)	0.65 (0.61,0.68)	0.6 (0.56,0.63)
	**F1**	0.62 (0.58,0.65)	0.55 (0.52,0.59)	0.57 (0.54,0.61)	0.57 (0.54,0.61)	0.66 (0.58,0.74)	0.56 (0.52,0.6)	0.62 (0.58,0.66)	0.57 (0.54,0.61)
	**Precision**	0.65 (0.61,0.69)	0.59 (0.54,0.65)	0.59 (0.55,0.63)	0.6 (0.56,0.64)	0.69 (0.6,0.78)	0.59 (0.53,0.64)	0.66 (0.62,0.71)	0.61 (0.55,0.63)
	**Recall**	0.64 (0.6,0.67)	0.61 (0.58,0.63)	0.59 (0.56,0.63)	0.6 (0.56,0.63)	0.67 (0.6,0.75)	0.61 (0.58,0.64)	0.65 (0.61,0.68)	0.61 (0.56,0.63)

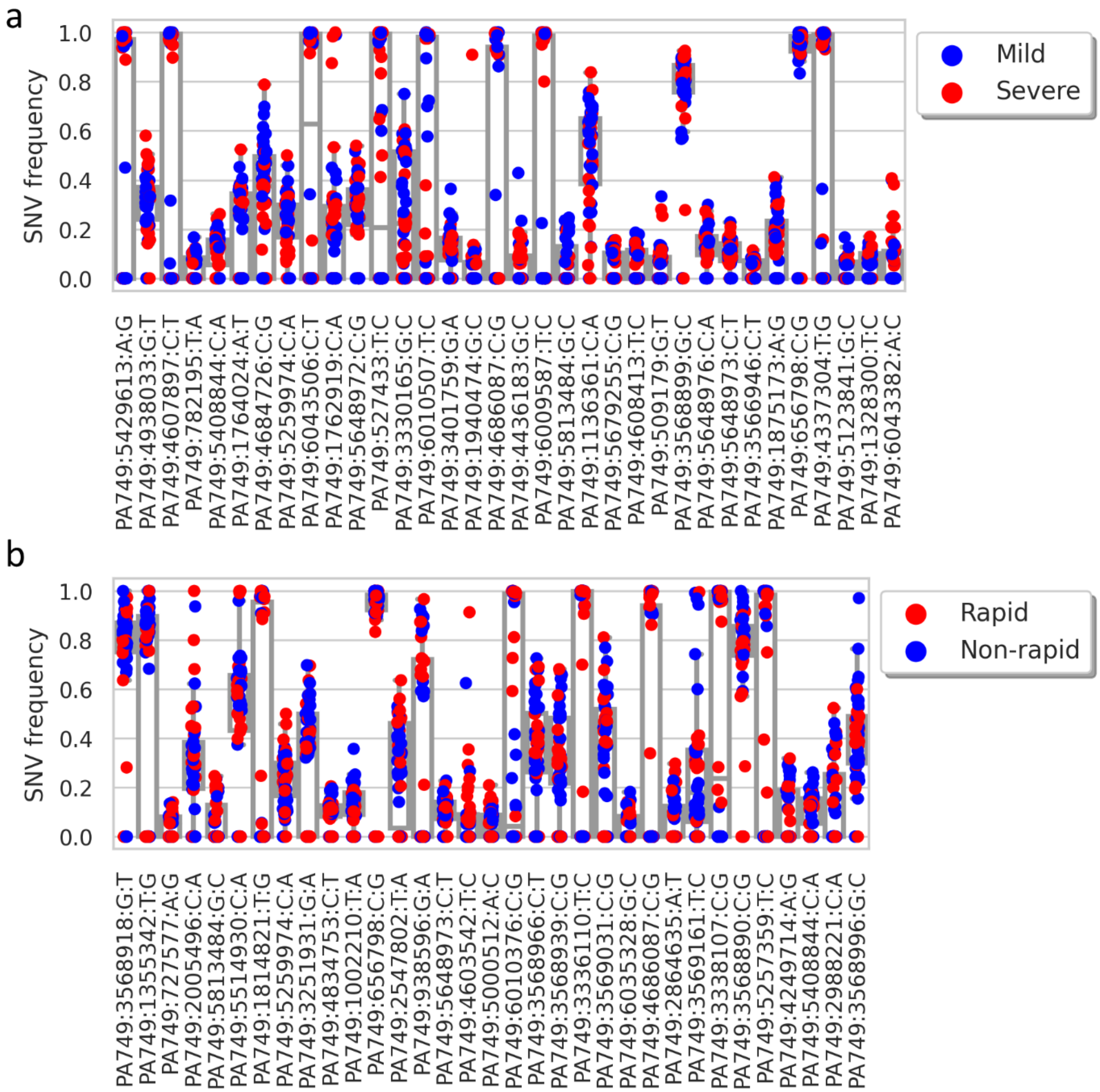

Supplementary Figure 1. Distribution of predictor SNV frequencies.

Frequencies of identified predictor SNVs for (A) baseline lung function and (B) lung function decline across 54 patients. Samples are color-coded based on lung disease condition (Methods). Predictor SNVs are ordered based on their estimated feature importance (from high on the left, to low on the right) on the x-axis.

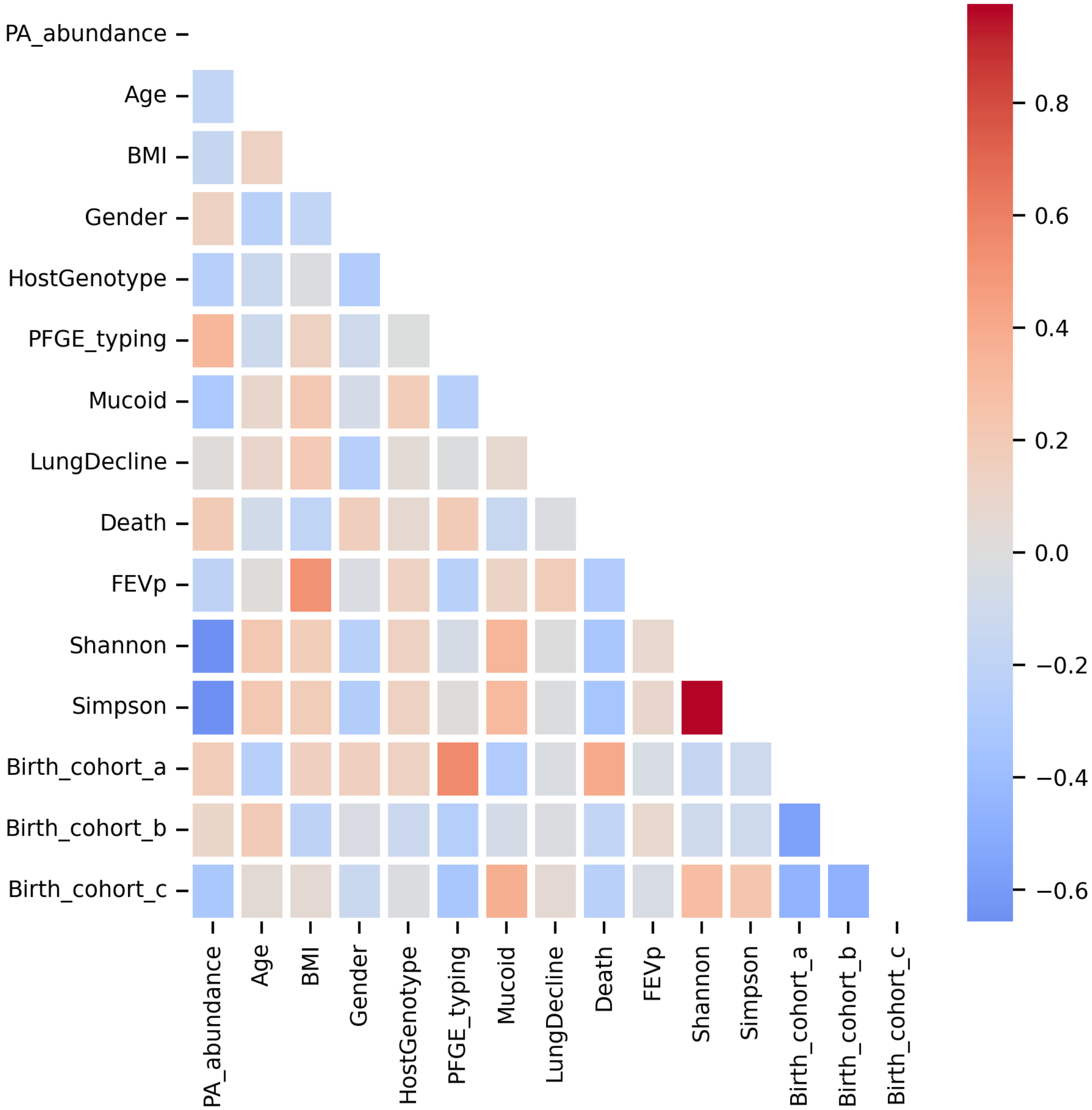

Supplementary Figure 2. Correlation between the evaluated clinical factors.

Pairwise Pearson correlation coefficients (R^2) heatmap of clinical data used prediction of lung disease severity and progression in CF patients.

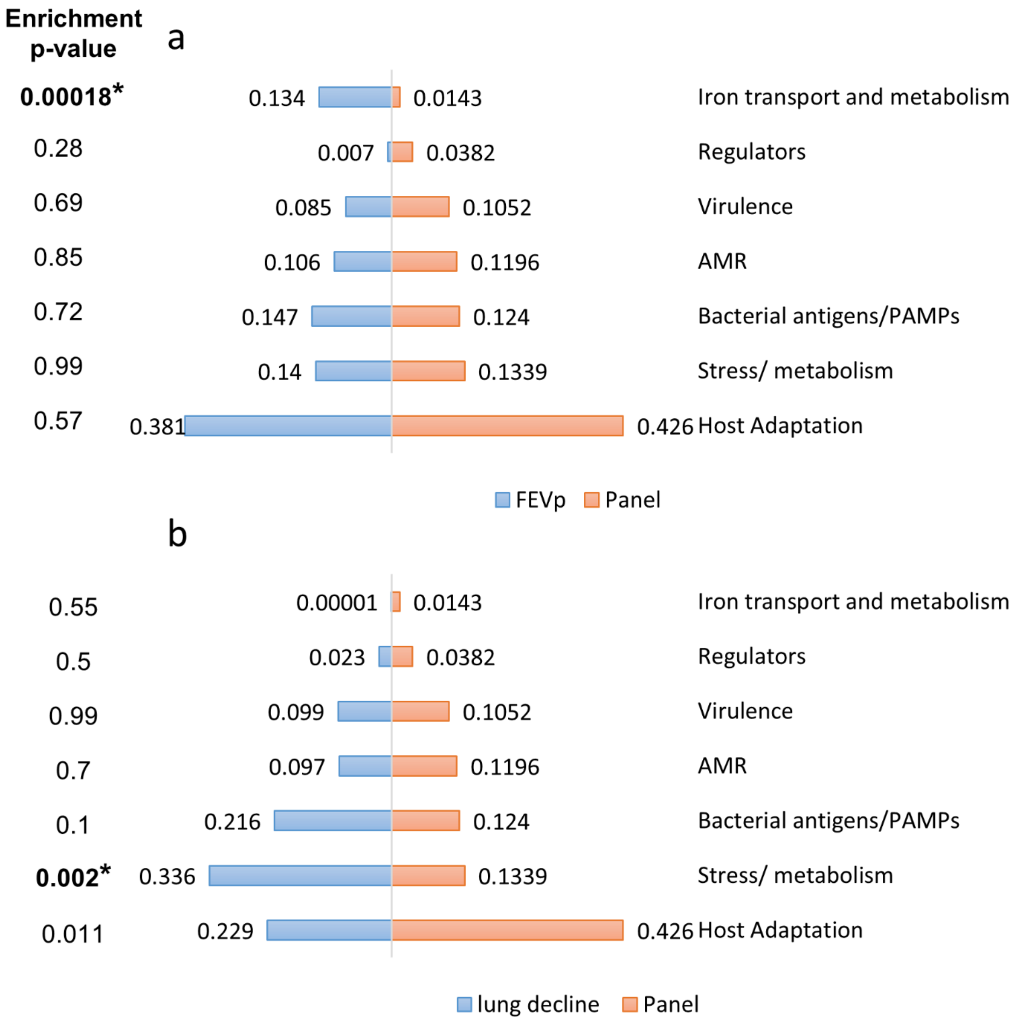

Supplementary Figure 3. Enrichment of predictor SNVs across functional categories in the AmpliSeq panel.

Predictor SNVs were functionally classified based on the function of genes harboring them (i.e. predictor genes) and enrichment of predictor genes for (a) baseline lung function (FEVp) and (b) progression (lung function decline) relative to total genes included in the AmpliSeq panel. Categories enriched in predictor genes relative to their expected frequency in the panel were identified using Fisher's Exact test. Statistically significant categories were determined at a significance level of $P < 0.05$ adjusted for multiple testing using the Bonferroni method (*i.e.* unadjusted $P < 0.005$).

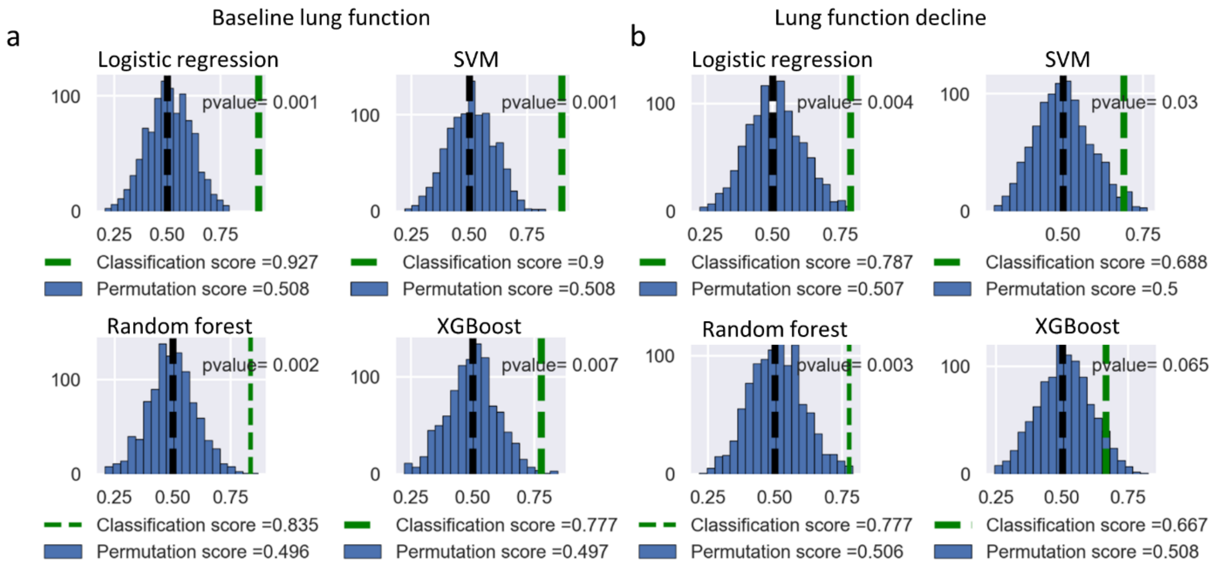

Supplementary Figure 4. Statistical significance of the performance of different ML models for prediction of lung disease severity and progression.

Average AUROC (area under the receiver operating characteristic curve) scores of four different ML models for (A) baseline lung function, and (B) lung function decline prediction compared with the same dataset with randomly shuffled labels.

(a)

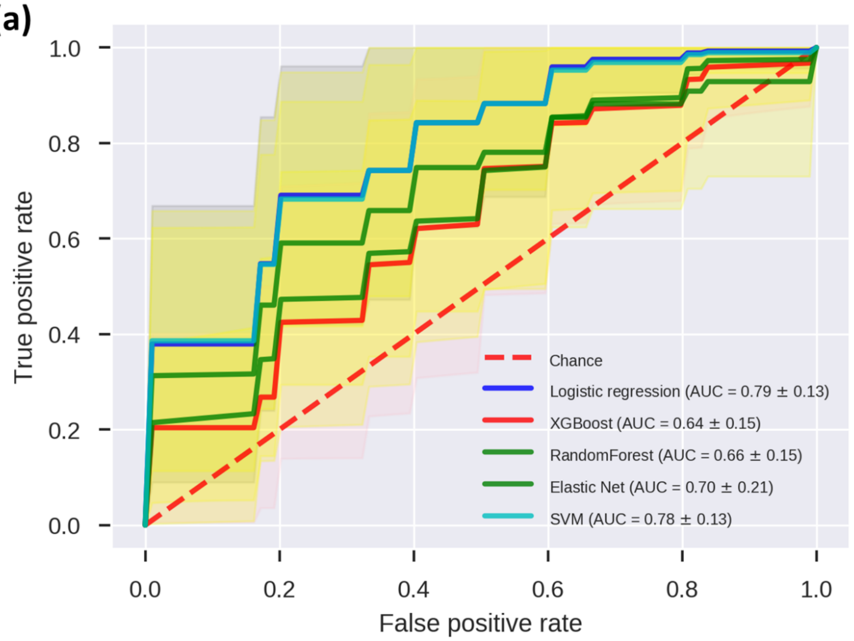

(b)

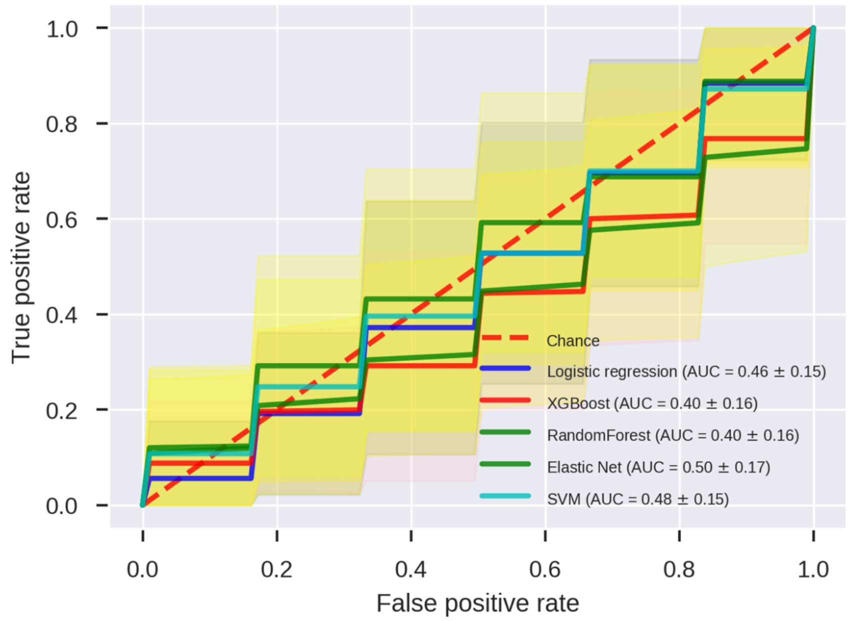

Supplementary Figure 5. Predictive models of baseline lung function and lung function decline based on clinical factors alone.

a) Average AUROC scores of different ML models to classify baseline lung function using clinical data compared to the random expectation (red dashed line). Shading indicates the 95% confidence interval.

b) Average AUROC scores for lung function decline prediction. Predictions are not significantly better than random.

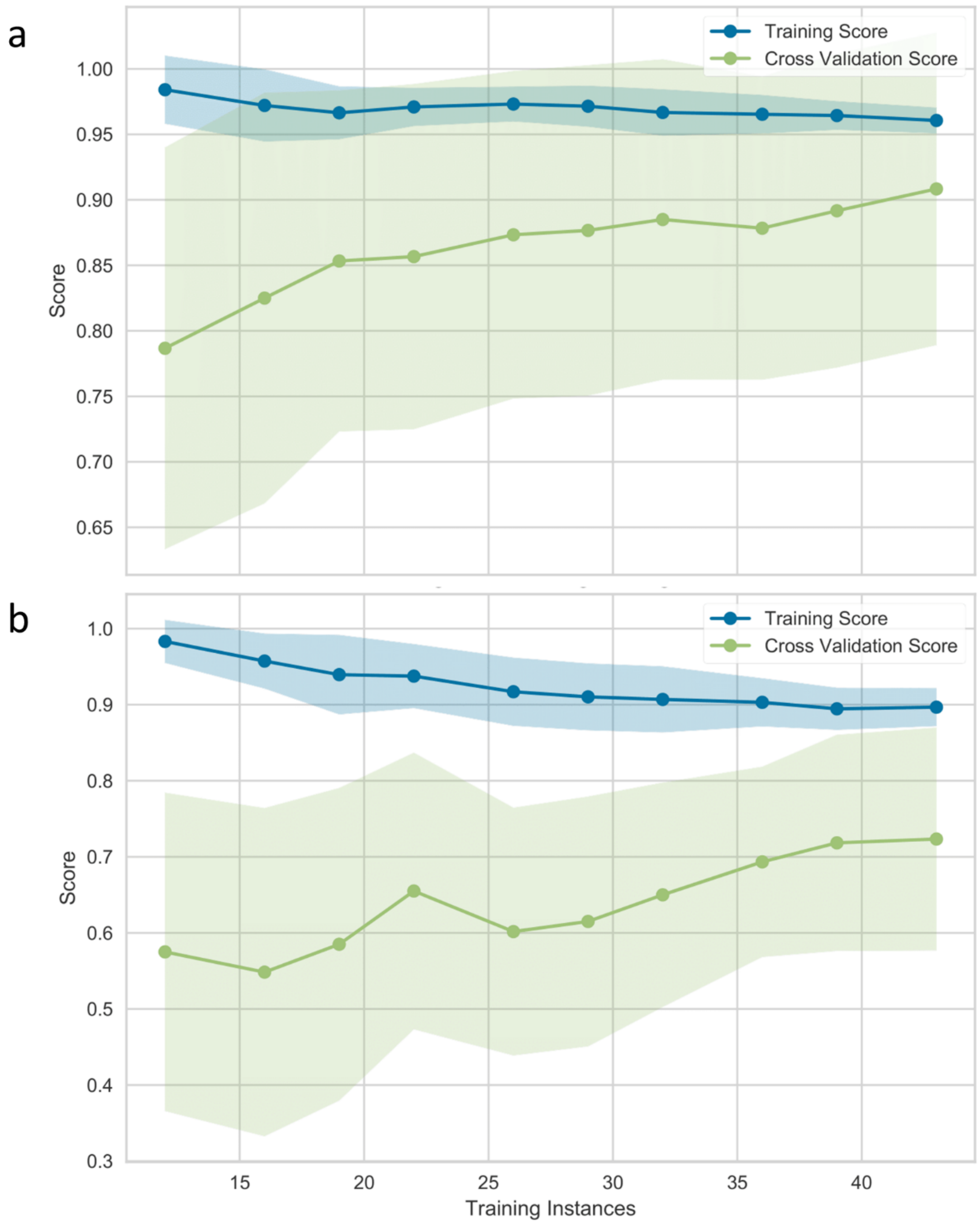

Supplementary Figure 6. Learning curve of logistic regression models for prediction of lung disease severity and progression.

Learning curves show the change in mean training accuracy (blue line) and cross-validation accuracy (green line) in predicting (A) baseline lung function, and (B) lung function decline as increasing numbers of samples are used to train the logistic regression model. Shading indicates the 95% confidence interval. Assessments at each number of training examples were through 20-fold stratified shuffled cross-validation.
